# Leukocyte Response to *Campylobacter* Intra-Abdominal Infection in One Day Old Leghorn Chickens

**DOI:** 10.3390/microorganisms11030613

**Published:** 2023-02-28

**Authors:** Kenneth J. Genovese, Haiqi He, Christina L. Swaggerty, J. Allen Byrd, Michael H. Kogut

**Affiliations:** Food and Feed Safety Research Unit, Southern Plains Agricultural Research Center, U.S. Department of Agriculture, Agricultural Research Service, College Station, TX 77845, USA

**Keywords:** *Campylobacter*, poultry, leukocyte, heterophil, immune response

## Abstract

Using a previously characterized and described abdominal model to define the avian immune response to *Salmonella* intra-abdominal challenge in chickens, we have adapted this technique for the study of chickens’ immune response to a *Campylobacter* intra-abdominal challenge. The intra-abdominal *Campylobacter* infection model facilitates the characterization of peripheral blood leukocyte dynamics and abdominal cell infiltrates. Day-of-hatch Leghorn chickens were injected intra-abdominally (IA) with *Campylobacter jejuni* [(CJ)1 × 10^8^ colony-forming units (CFUs)]. Changes in peripheral blood leukocyte numbers and abdominal cell infiltrates were monitored at 0, 4, 8, and 24 h post-injection. Peripheral blood leukocyte numbers were also determined for 2 h post-injection. For mortality studies, birds were injected intra-abdominally with 1 × 10^8^ CFUs CJ and mortalities were recorded for 72 h post-injection. In the peripheral blood of CJ-injected chicks, total white blood cell (WBC) numbers began increasing by 2 h post-injection, peaking at 4 h post-injection with the predominant cell type being polymorphonuclear leukocytes (heterophils). Total WBCs declined after 8 h and this decline continued at 24 h, with total WBC numbers approaching control values. The injection of CJ into the abdominal cavity caused a rapid rise in abdominal cell infiltrates with the predominant infiltrating leukocytes being heterophils. Peak abdominal heterophil infiltrates were observed at 8 h post-injection, declining only slightly by 24 h post-injection. Mortality in the CJ challenge groups reached 37%. Mortality in the *Salmonella enteritidis* positive control groups were greater than 50%. The data suggest that *Campylobacter* infection does stimulate the innate immune response in chickens when administered IA, however, the immune response and infection is not characterized with the high levels of mortality observed with a *Salmonella* infection. These data provide a basis for a more definitive characterization of chickens’ immune response to *Campylobacter* and a model to evaluate intervention strategies to prevent the infection and colonization of poultry.

## 1. Introduction

*Campylobacter* infections in humans linked to uncooked or improperly handled poultry products are a major public health concern. The World Health Organization considers *Campylobacter* one of four key global causes of diarrheal disease [1]. The Centers for Disease Control (CDC) estimates that 2.4 million people have diseases associated with *Campylobacter* each year and approximately 124 people die from the disease [2]. *Campylobacter* and its effects on the host have been extensively observed in humans due to the disease that it causes. In its animal hosts (swine, poultry, cattle, etc.), the effects of *Campylobacter* on immune function have begun to be more thoroughly studied.

Data from previous studies using an intra-abdominal (IA) model of *Salmonella enteritidis* (SE) infection show that SE causes changes in peripheral blood leukocyte numbers and causes an inflammatory cell influx into the abdominal cavity, normally a cell-free location in the body [3]. Heterophil influx into the abdominal cavity was found to increase significantly at 4 h post-infection [3]. Studies in turkeys also show SE causing changes in peripheral blood leukocyte numbers after oral administration of SE [4]. Meade and colleagues described the effects of *Campylobacter* on a chicken’s immune system after an oral challenge at four weeks of age and found that *Campylobacter* had no effect on circulating heterophil numbers but did cause an early rise in peripheral blood monocytes/macrophage [5]. In addition, toll-like receptor (TLR) 21 expression was found to increase and antimicrobial peptide gene expression was found to decrease [5]. In the present study, an intra-abdominal (IA) model of *Campylobacter* infection was used in day-old Leghorn chicks to test the effects on peripheral blood leukocyte numbers and chick mortality.

## 2. Materials and Methods

### 2.1. Animals

Day-of-hatch male Leghorn (Hy-Line W-36^®^) chicks were obtained from a commercial hatchery (Hy-line International, Bryan, TX, USA). Total number of chicks used for peripheral blood counts: 90 chicks per group over three repetitions; 90 chicks per group over three repetitions for abdominal lavage studies. Total number of chickens used in three repetitions in mortality studies: control 220; CJ 205; SE 200. Chicks were placed into floor pens with wood shavings for bedding and had free access to feed and water. The feed followed the nutritional recommendations of the NRC 7 (National Research Council, 1994). Additional warmth was provided by heat lamps. All protocols involving animals were reviewed and accepted by the Animal Care and Use Committee, USDA, ARS, SPARC.

### 2.2. Campylobacter Jejuni (CJ)

A poultry field isolate of CJ was obtained from Dr. J. Allen Byrd, USDA, ARS, SPARC. CJ was grown in Mueller Hinton broth (MHB) for 48 h at 42 °C prior to challenge. The CJ was washed once in PBS. CJ was resuspended in the same volume of PBS that it was grown in. Birds were then dosed with this concentration of CJ (0.5 mL/bird). The CJ challenge was diluted and plated (MH plates) to verify the CJ challenge dose per bird.

### 2.3. Salmonella Enteritidis (SE)

A poultry isolate of SE #97-11771 was obtained from the National Veterinary Services Laboratory (Ames, IA). SE was cultured in tryptic soy broth (TSB) overnight at 41 °C. The bacteria were pelleted (7700 g for 10 min) and washed with ice-cold phosphate-buffered saline (PBS), centrifuged at 7700× *g* for 10 min, supernatant discarded, and the pellet re-suspended in 1 mL cold PBS and diluted to 1 × 10^8^ colony-forming units (cfu)/mL in PBS using a Spectronic 20D spectrophotometer (Milton Roy Co., Golden, CO, USA) with a 625 nm reference wavelength.

### 2.4. Peripheral Blood Counts

Five chicks from each of the treatment groups described above were randomly selected at 0, 2, 4, 8, and 24 h post-injection and 0.1 mL of blood from each chick was collected in a capillary tube and placed in 1.9 mL of chicken blood diluent [6]. Total leukocytes were counted using a hemocytometer. At the same time, blood smears were prepared, air-dried, and stained using the Hema 3 system (Curtin Matheson Scientific Co., Houston, TX, USA). Cell counts of lymphocytes, large mononuclear cells (LMNs, monocytes, blast cells), and PMNs (heterophils) were made microscopically using oil immersion. The number of heterophils per mm^−3^ were calculated for each chick from total and differential leukocyte counts.

### 2.5. Abdominal Lavage and Cell Infiltrates

Day-old chicks were randomly placed in control and treated groups and were maintained under the conditions described above. Day-of-hatch Leghorn chickens were injected intra-abdominally with *Campylobacter jejuni* [(CJ)1 × 10^8^ colony-forming units (CFUs)]. From dilution of the 1 × 10^8^ CFU stock, SE groups received 5 × 10^3^ CFU/ bird by IA injection for both the mortality and leukocyte studies. Changes in peripheral blood leukocyte numbers and abdominal cell infiltrates were monitored at 0, 2, 4, 8, and 24 h post-injection. At time 0, a collection was performed on five chicks per repetition to establish a baseline of cell infiltrates into the abdominal cavity. At each collection point, five chicks were randomly selected from the respective experimental groups and euthanized by CO_2_ asphyxiation before their abdominal cavities were lavaged with a total of 2 mL of Ca^2+^—Mg^2+^-free Hank’s balanced salt solution containing 0.1 M disodium ethylene diamine tetra-acetic acid and 0.25% bovine serum albumin (Sigma Chemical Co., St. Louis, MO, USA). The abdominal wash from each chick was maintained separately. The recovered IA cell infiltrates were enumerated as described above. A cytospin smear was prepared for each chick at each time point and stained, and fixed and differential leukocyte counts were performed for each chick IA exudate sample as described earlier. Peripheral blood and IA cell infiltrate studies represent three repetitions (10 birds per group/time point/repetition).

### 2.6. Mortality Evaluation

Chicks were injected intra-abdominally with 1 × 10^8^ CFUs CJ; 1 × 10^8^ SE and mortalities were recorded for 72 h post-injection. Total number of chickens used in three repetitions in mortality studies: control 220; CJ 205; SE 200. Birds that succumbed in the first 4 h post-infection were not considered CJ- or SE-associated mortalities. Birds whose condition provoked humane euthanasia were considered mortalities for all groups after the first 4 h post-infection.

### 2.7. Statistical Analysis

Statistical analysis was performed using SigmaStat^®^ statistical software (Jandel Scientific, San Rafael, CA, USA). Differences between the two experimental groups and time points were determined using ANOVA + Tukey (*p* ≤ 0.05).

## 3. Results

### 3.1. Peripheral Blood Leukocytes

**Figure 1:**
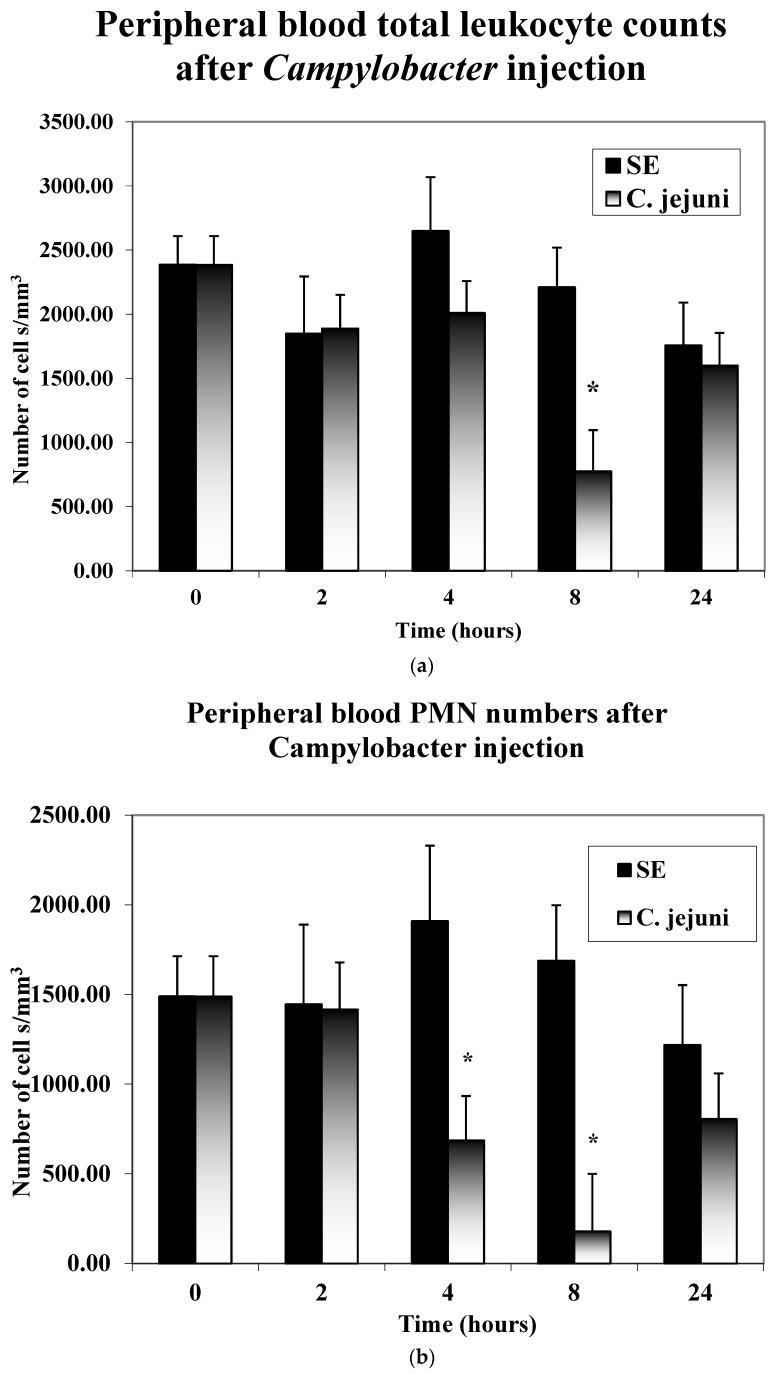
(**a**,**b**) Peripheral blood counts. Five chicks from each of the treatment groups described above were randomly selected at 0, 2, 4, 8, and 24 h post-injection. Blood smears were prepared. Leukocyte cell counts of lymphocytes and large mononuclear cells (LMNs, monocytes, blast cells, heterophils) were made microscopically using oil immersion. The numbers of heterophils per mm^−3^ were calculated for each chick from the total and differential leukocyte counts. Significant differences are indicated with an asterisk * (*p* ≤ 0.05).

As seen in Figure 1a,b, the number of leukocytes in the peripheral blood decreased significantly at 8 h post-injection in the CJ-injected birds. In particular, the number of heterophils in the peripheral blood were observed to decrease at 4 h post-injection, which continued at 8 h post-injection, then increased at 24 h post-injection. *Salmonella*-injected birds showed a marked increase in peripheral blood heterophils at 4 and 8 h post-injection, then decreasing at 24 h post-injection.

### 3.2. Abdominal Cell Infiltrates

**Figure 2:**
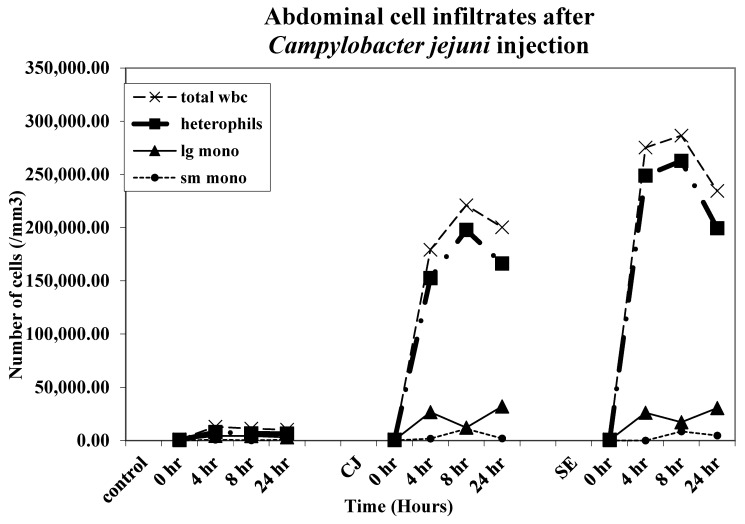
Abdominal cell infiltrates. Chicks were injected IA with *Campylobacter jejuni* [(CJ)1 × 10^8^ colony-forming units (CFUs)]. SE groups received 5 × 10^3^ CFU/ bird. Abdominal cell infiltrates were monitored (five chicks/group/timepoint) at 0, 2, 4, 8, and 24 h post-injection. The recovered IA cellular infiltrates were counted on a hemocytometer as described above. A cytospin smear was prepared for each chick at each time point and stained, and fixed and differential leukocyte counts were performed for each chick IA exudate sample as described earlier. Peripheral blood and IA cell infiltrate studies represent three repetitions (15 birds per group/time point/repetition).

All leukocyte types, heterophils, large monocytes, and small monocytes were found to infiltrate the abdominal cavity after injection with CJ and SE (Figure 2). The increase in heterophil cell infiltrates in both the CJ and SE groups began at 4 h post-injection, peaked at 8 h post-injection, and began declining at 24 h post-injection. The increase in heterophil infiltration was greater in the SE group compared to the CJ group.

### 3.3. Mortality

**Figure 3:**
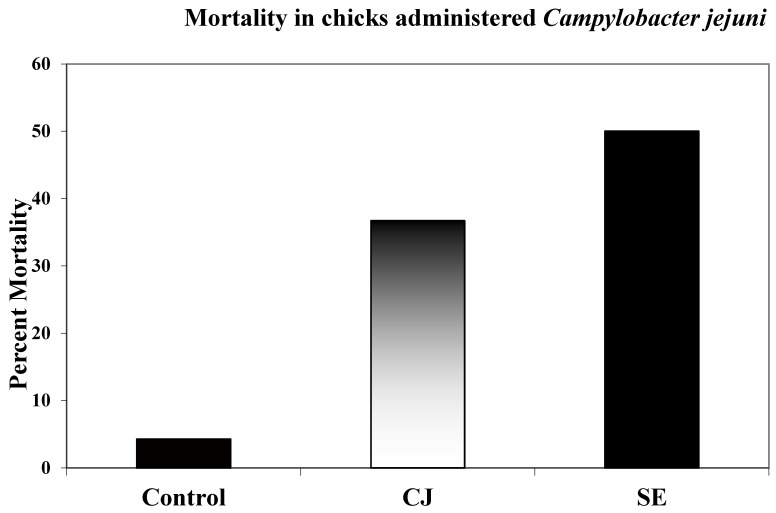
Chick mortality. Birds were injected intra-abdominally with 1 × 10^8^ CFUs CJ; 1 × 10^3^ SE and mortalities were recorded for 72 h post-injection. Total numbers of chickens used in three repetitions in mortality studies: control 220; CJ 205; SE 200. Birds that succumbed in the first 4 h post-infection were not considered CJ- or SE-associated mortalities. Birds whose condition provoked humane euthanasia were considered mortalities for all groups after the first 4 h post-infection.

Total mortality in all groups was recorded over a 72 h period. Birds that succumbed in the first 4 h after injection were not recorded as associated with CJ or SE injection. Total mortality over 72 h in the CJ group was 37% while mortality in the SE group was 50% (Figure 3).

## 4. Discussion

*Campylobacter* remains one of the most important foodborne pathogens in modern food animal production systems. Poultry constitute a large portion of cases of foodborne illness associated with *Campylobacter*. The colonization of a chicken’s intestinal tract with *Campylobacter* does not appear to be detrimental to the chicken and is considered by many as a commensal organism in the GIT tract of poultry and other species [7,8].

The present study found that the IA injection of CJ in chickens resulted in fewer heterophils in the peripheral blood at 4, 8, and 24 h post-injection as compared to SE-injected control birds. Total leukocytes in the peripheral blood were also found to be reduced as compared to the SE-injected control birds at 4 and 8 h post-injection. Abdominal cell infiltrates in the CJ-injected group followed a similar time course pattern as those in the SE-injected group. However, the number of leukocyte infiltrates into the abdomen were reduced compared to the SE-injected control birds. In addition, chicken mortality post-injection was found to be less in the CJ-injected group compared to the SE-injected group (37% vs. 50%, respectively).

In theory, the decrease in heterophils in the CJ-injected group in the peripheral blood would be expected due to an influx of heterophils in the abdominal cavity. However, this trend is not seen in *Salmonella*-injected birds. Both in the peripheral blood and abdominal cavity, large increases in heterophils are observed in *Salmonella*-injected birds. The total leukocyte counts in the abdomen and peripheral blood are higher in the *Salmonella*-injected group. The results would appear to point to a much more robust immune response of the host to *Salmonella* injection compared to CJ injection. The current literature on *Campylobacter* and the immune response of chickens appear to support this observation [9,10,11].

The data presented, along with data in the literature, paint a picture of *Campylobacter* as having host immune interactions that would point to a possible disease process in poultry. However, disease is not observed in naturally infected birds in commercial production systems. Meade and colleagues observed the immune response and leukocyte response in 4-week-old broiler chickens when challenged by the traditional route of oral gavage. By oral challenge, peripheral blood heterophil numbers were found to be unaffected, whereas monocyte numbers were found to increase early on after challenge [5]. The peripheral blood changes observed in the present study associated with *Salmonella* administration were also observed in 4-week-old broilers upon oral challenge with *Salmonella* [5]. There may be multiple issues presented by the *Campylobacter* challenge findings as they are dissimilar to those in the present studies. The use of day-old laying chickens in the study presented here, as well as the route of administration, may account for some differences. Researchers have found that challenging birds prior to three weeks of age with *Campylobacter* may be inhibited or reduced by maternal antibodies [12]. After three weeks of age, MAB in chicks has diminished to a point where colonization of the chicks’ GI tract with *Campylobacter* is unfettered and can reach the 100% colonization of birds in commercial settings [12]. Deng et al. have noted that resistance to colonization of the GI tract in chickens is highest in birds from 1 to 2 weeks of age [11]. Also noted was that different breeds and lines of chickens responded differently to *Campylobacter* challenge, finding that in slower growing breeds, IL-10 mitigates an inflammatory response to *Campylobacter* as compared to faster growing breeds which do not produce similar levels of IL-10, with subsequent ongoing inflammation and diarrhea in the latter [12,13,14,15]. Meade et al. also found that chickens given *Campylobacter* orally at 4 weeks of age exhibited a decrease in the gene expression of antimicrobial peptides [5,16]. Taken together, it would appear *Campylobacter* can cause disease in chickens, perhaps depending on the breed of chicken, but most research points to an altered/decreased immune response to *Campylobacter* when compared to the immune response elicited by *Salmonella* in chickens.

The main goal of this study was to test *Campylobacter* IA injection and compare it to the results of SE IA injection. We have previously used the IA model to investigate the effects of an IA injection of *Salmonella* on the host, as measured by cell influx into the abdominal cavity and peripheral blood white blood cell dynamics and the use of immune stimulating agents to reduce subsequent mortality and infection in chickens [3,4]. The IA model allows for an observation of the effects of the bacteria on host immunity without the added caveat associated with an oral challenge and unavoidable interactions with the GI flora. Due to the limited nature of the studies presented here, the conclusions made must be narrow in focus. The studies show that *Campylobacter* administered in the IA model does elicit a cellular response in the abdominal cavity and peripheral blood, and mortality associated with an IA injection is reduced compared to an injection of *Salmonella*. Further studies designed to achieve a larger, more intricate measurement and characterization of the immune response are planned.
